# Understanding Attitudes towards Proenvironmental Travel: An Empirical Study from Tangshan City in China

**DOI:** 10.1155/2014/963683

**Published:** 2014-11-04

**Authors:** Xiaoping Fang, Yajing Xu, Weiya Chen

**Affiliations:** School of Traffic and Transportation Engineering, Central South University, Changsha 410075, China

## Abstract

Understanding people's attitudes towards proenvironmental travel will help to encourage people to adopt proenvironmental travel behavior. Revealed preference theory assumes that the consumption preference of consumers can be revealed by their consumption behavior. In order to investigate the influences on citizens' travel decision and analyze the difficulties of promoting proenvironmental travel behavior in medium-sized cities in China, based on revealed preference theory, this paper uses the RP survey method and disaggregate model to analyze how individual characteristics, situational factors, and trip features influence the travel mode choice. The field investigation was conducted in Tangshan City to obtain the RP data. An MNL model was built to deal with the travel mode choice. SPSS software was used to calibrate the model parameters. The goodness-of-fit tests and the predicted outcome demonstrate the validation of the parameter setting. The results show that gender, occupation, trip purpose, and distance have an obvious influence on the travel mode choice. In particular, the male gender, high income, and business travel show a high correlation with carbon-intensive travel, while the female gender and a medium income scored higher in terms of proenvironmental travel modes, such as walking, cycling, and public transport.

## 1. Introduction

Climate change is a huge challenge that humans must confront, and CO_2_ emissions must be reduced to mitigate global warming [1]. Therefore, carbon reduction is becoming an important proenvironmental target of every country. The transportation industry accounts for nearly one-quarter of the total carbon emissions all over the world, while carbon emissions from cars account for three-quarters of the total carbon emissions in the transportation industry [2]. Thus, cars are a high-carbon travel mode. Since the carbon emissions from public transportation are far below those of cars, we can refer to public transportation as a low-carbon travel mode. Other travel modes, such as walking and cycling, have almost no carbon emissions, so they are called zero-carbon travel modes. Therefore, walking, cycling, and public transportation, which are energy saving, oppose pollution, and generate low levels of carbon emissions, are deemed to be proenvironmental travel modes.

Giving priority to the development of public transportation and encouraging the public to choose a proenvironmental travel mode have become many countries' sustainable transportation development strategies. The Government of Great Britain emphasizes its low-carbon-oriented policies in its “Low Carbon Transport Innovation Strategy” by giving priority to the development of public transportation, constructing slow traffic and public bike systems and encouraging walking, cycling, public transportation, and other noncarbon or low-carbon transportation [3]. A number of environmental, educational, and comprehensive intervention programs have been conducted by many countries in the past decades to promote citizens' voluntary proenvironmental travel. China has also promoted a public transportation development strategy as a national strategy. In many cities, urban public transit, public bicycles, and slow tracks have been developed rapidly. In some cities, members of the public are even encouraged by subsidies to travel by public transport. To build an effective public transportation system, conduct effective education, and adopt intervention strategies to promote voluntary proenvironmental travel, we must first understand the extent to which factors can influence the public to choose a proenvironmental travel mode in China. In this paper, based on Samuelson's theory of consumer choice and preference relations [4], we choose a medium-sized city—Tangshan—to conduct a revealed preference investigation based on the following reason. The average travel distance is very long and very serious traffic jam is often seen in ground transportation system in megacities like Beijing, Shanghai, and Guangzhou. Many people choose subway involuntarily to a large extent in these big cities. Tangshan is a middle-sized city in China. The average urban travel distance is much shorter than that of big cities. There is not much traffic jam. This paper studies the influencing factors of voluntary proenvironmental travel. We believe that such a middle-sized city would be a better sample.

## 2. Theoretical Background

### 2.1. Travel Mode Choice Decision Theory

Determinants of behavior include motivation and will [5], which have been proved by the theory of reasoned action [6, 7]. Over the past decade, the study of proenvironmental travel behavior psychology has essentially been based on two theories [8]: the theory of planned behavior (TPB) [9] and norm activation theory [10]. It is proposed that, to achieve large-scale changes in travel behavior, it is important to change carbon-intensive travel habits [11]. Therefore, many researchers are committed to exploring the extent to which changing the travel-related costs, benefits, and alternatives can break car use habits [12]. The norm activation theory, originally used to explain prosocial behavior, has lately been developed into the value-faith-gauge theory [13], which explains car user education better than the TPB [14]. Attitude-context-behavior (ACB) is a predicting environmental behavior theory developed on the basis of the acts model of Lewin by Guagnano et al. [15]. They found external situational impact factors and verified the rationality. In recent years, a comprehensive analysis named the Scheme for the Comparative Analysis of Public Environmental Decision-Making (SCAPE) has been developed [16]. The basic idea is that since we cannot understand every person exactly, why not take individuals as a black box with a system opinion and build a model based on input and output to identify the relationship between behavior and motivations?

### 2.2. Influencing Factors of Travel Mode Choice

Understanding the influencing factors of proenvironmental travel behavior and their individual influencing path is the prerequisite for the scientific development of transportation decision-making and intervention strategies. The factors that influence proenvironmental travel can be divided into individual characteristics, social characteristics, and situational variables.

In practice, behavioral change theory prevails to promote the voluntary reduction of car use and a shift to proenvironmental travel, of which some classic economic indicators (such as prices and taxes) and land use, transportation network, and behavior-oriented traffic systems have always been welcomed [17, 18]. Understanding individual travel decision making is considered the key to promoting large-scale change in proenvironmental travel by public policy and education. The quality of the public transport service, travel characteristics, and personal characteristics are confirmed to exert a significant impact on travel choices [19].

Researchers usually choose factors (variables) according to the purpose of specific research topic and considering the difficulties in data collecting. In this paper, we mainly considered the following aspects when selecting variables as done in similar research: (1) the variables group should effectively express the main relationship between travel decision and factors; (2) when satisfying the first consideration, a feasible and economical investigation should be taken into our consideration.

## 3. Model

Traditionally, aggregate models were always used to study the influence of psychological variables, but when attempting to investigate the influence of some situational factors, a disaggregate model will be more appropriate. The main reason is that the situational factors can be measured although they are varied, while psychological variables cannot be measured directly, although they are relatively stable [20]. The basic assumption of the disaggregate model is that when travelers are faced with travel mode choices, the “utility value” of choices can be used to describe travelers' preference for each travel mode. Utility is the function of the selected object's properties and the decision makers' characteristics. The disaggregate model is based on utility maximization theory and random utility theory. Compared with the aggregate model, the disaggregate model has the advantage of strong portability and multiple variables.

McFadden innovatively introduced the “utility theory” of economics into transportation and proposed a new logit mode called the “random utility model” [21, 22]. Domencich presented a discrete choice model based on “maximum utility theory” and then further divided the disaggregate model into the logit model family and the probit model family, based on which a theoretical system of the disaggregate model was gradually formed [23]. Ben-Akiva, Lerman, and Vovsha further introduced the theory into traffic demand forecasting, conducting deep research into the transportation division problem and pushing the logit model into the practical application stage [24, 25]. By analyzing individuals' unobserved and observed preferences and characteristics, Bhat used the multinomial logit model (MNL) to describe the personal preference for transportation and analyzed individuals' travel mode choice behavior under different service levels [26]. In economics, it is assumed that consumer preferences can be represented by a continuous utility function, which can be mathematically proved. According to random utility theory, travelers will choose the travel mode at their perceived maximum utility in a specific situation.

According to random utility theory, the utility function *U* consists of nonrandom and random parts as follows:

(1) $$\begin{array}{c} U_{in}=V_{in}+\varepsilon_{in}, \end{array}$$

where *U*_*in*_ is the utility function of the alternative travel mode *i*  (*i* = 1,2,…, *J*) of traveler *n*  (*n* = 1,2,…, *N*); *V*_*in*_ is the nonrandom part of the utility function; and *ε*_*in*_ is the random part of the utility function, which are submitted to Gumbel distribution and independent from each other.

Traveler *n* would choose *i* if and only if

(2) $$\begin{array}{c} U_{in}>U_{jn},\;i\neq j,\;\;i,j\in A_{n}, \end{array}$$

where *A*_*n*_ is the set of all possible travel mode choices of traveler *n*.

According to maximum utility theory, the probability that traveler *n* will choose travel mode *i* is denoted as *P*_*in*_ as follows:

(3) Pin=ProbUin>Ujn;i≠j, i,j∈An=ProbVin+εin>Vjn+εjn;i≠j, i,j∈An,

where 0 ≤ *P*_*in*_ ≤ 1, ∑_*i*∈*A*_*n*__ *P*_*in*_ = 1.

## 4. Data and Application

### 4.1. Sample, Predictor, and Data Processing

This paper chooses Tangshan as the sample city. Tangshan is a medium-sized city located in North China, the economic development level, city size, and traffic conditions of which are in the intermediate state. There is no subway in Tangshan, and motorcycles have been banned from the urban district. The set of alternative travel modes available for residents is denoted as *A*:

*A* = {*i*∣*i* = 1, walking; *i* = 2, bicycle; *i* = 3, electric  bicycle; *i* = 4, bus;  *i* = 5, taxi; *i* = 6, private  car}.

Field investigation by questionnaire survey is conducted to find the factors affecting the travel mode choice. Thirteen possible factors of personal characteristics, family-owned private travel tool characteristics, and travel characteristics are the assumed variables (*k* is the number of variables; *k* = 1,2,…, *K*, *K* is the total number of variables), which are presented in Table 1.

We surveyed 450 respondents in central business districts, outlets, transportation hubs, office buildings, and large enterprises in Tangshan. Out of the total of 424 questionnaires received, 419 are qualified. The calculation is executed by SPSS software.

### 4.2. Utility Function

The MNL model is used to model the individual travel mode choice. It is assumed that all the factors are independent from each other and obey the Gumbel distribution with zero mean. Equation (4) is the utility function:

(4) $$\begin{array}{c} V_{in}=\theta_{i}X_{in}=\theta_{i0}+\overset{K}{\underset{k=1}{\sum }}\theta_{ik}X_{ink},\;\left(i\in A_{n}\right). \end{array}$$

In (5), *P*_*in*_ is the probability of traveler *n* selecting travel mode *i*:

(5) $$\begin{array}{c} P_{in}=\frac{\exp\left(V_{in}\right)}{\sum_{j\in A_{n}}\exp\left(V_{jn}\right)}=\frac{\exp\left(\theta_{i}X_{in}\right)}{\sum_{j\in A_{n}}\exp\left(\theta X_{jn}\right)},\;\left(i\in A_{n}\right), \end{array}$$

where *V*_*in*_ is the utility function when traveler *n* chooses travel mode *i*; **X**_**in**_ = [*X*_*in*0_, *X*_*in*1_,…, *X*_*ink*_,…, *X*_*inK*_] is an eigenvector of traveler *n* choosing travel mode *i*; the component *X*_*ink*_ is the value of variable *k* when traveler *n* chooses mode *i*, *X*_*in*0_ = 1; ***θ***_**i**_  =  [*θ*_*i*0_, *θ*_*i*1_,…, *θ*_*ik*_,…, *θ*_*iK*_] is the vector of utility coefficients; and *θ*_*ik*_ is the impact coefficient of variable *k* on travel mode *i*.

### 4.3. Results and Model Validation

SPSS17.0 is used to process the data. The results of the MNL model are shown in Table 2.

The calculated parameters in Table 2 and the variable values in Table 1 are put into (4) and (5) to calculate the utility value and choice probability. Therefore, it is possible to forecast the sample individuals' choice. The observed and forecasted choices are presented in Table 3.

There are different tests for model validation, the main ones of which are the goodness-of-fit test,* F*-test, and* t*-test. These three methods are fitted to test the linear model. Because the MNL model is a nonlinear exponential model and the unbiased estimate of the error variance cannot be obtained from the estimated residuals, the* t*-test or* F*-test cannot be used here to test the significance either for the individual or for the population [27]. Furthermore, the model residuals do not necessarily sum to zero and* ESS* and* RSS* do not necessarily add up to* TSS*; therefore, *R* ^2^ = *ESS*/*TSS* may not be a meaningful descriptive statistic for this model. Consequently, an alternative to pseudo* R*-square is proposed to estimate the goodness of fit. It can be seen as a rough approximation of model prediction accuracy [28]. Three pseudo* R*-squares calculated by SPSS are shown in Table 4. Generally, the pseudo* R*-squared value falls in [0, 1]. When the independent variable is completely unrelated to the dependent variable, the pseudo* R*-squared value will be close to zero; otherwise, it will be close to 1, which indicates that the model perfectly predicts the objective. The results listed in Table 4 show that the model is acceptable.

## 5. Analysis and Implication

### 5.1. Analysis

According to (4) and (5), the utility coefficients directly affect the value of the utility function, which can be used to decide the travel mode choice. The findings are presented below by comparing the utility coefficients of the different variables.

(*1) Gender*. It is noticed that the utility coefficient of males and females differs significantly among the alternatives. The differences in the three proenvironmental modes (walking, riding bicycle, and taking public transport) are much larger than those of carbon-intensive modes (taxi and private car); combined with that, the male utility values of all the travel modes are negative. This implies that men prefer carbon-intensive travel to proenvironmental travel, unlike women. The conclusion is in line with similar research [29] showing that women tend to adopt more socially responsible behavior.

(*2) Age*. The 20~50-year-old respondents prefer bicycles and private cars to electric bicycles, buses, and taxis, which indicates that there are two subgroups with different preferences in this group. The tendencies of the other groups are unclear. They may have a closer relationship with other characteristics.

(*3) Occupation*. First, it is noticed that civil servants extremely dislike electric bicycles and taxis. The Chinese Government had not strictly regulated official car use when the survey was conducted. If official cars were available freely, no civil servant would drive a private car, let alone ride an electric bicycle, which is more dangerous. Second, students show partiality for bicycles, taxis, and private cars. The choices of students are extremely different, which is related to their family status and the distance between school and home. Some students, whose home is very far away from school, usually lodge in the school or rent an apartment near school. The former mostly use taxis or private cars once a week, while walking or taking a bicycle is the best choice for the latter.

(*4) Family-Owned Private Travel Tools*. Only the utility coefficient of private cars is negative among all the travel modes of families that have no private car. Although they currently choose a proenvironmental travel mode, it does not explain whether their attitudes are proenvironmental. Their decisions probably change as soon as they own a private car.

(*5) Travel Distance*. When the distance is less than 1 kilometer, people tend to choose walking, a bicycle, or an electric bicycle; sometimes they also choose a private car, but seldom a bus or taxi.

(*6) Travel Purpose*. For commuting, the most frequent reason for travel, the utility coefficients of bicycles and electric bicycles are positive, while those of taxis and private cars are negative. The travel cost may explain the above choice, as well as some other factors, like the size of the city.

### 5.2. Implication

Public transportation plays a very important role in meeting the large travel demand and reducing carbon emissions. A high-level public transportation service is the premise for the public to choose a proenvironmental travel mode voluntarily. However, values and lifestyle provide the intrinsic motivation to engage in proenvironmental travel.

Our surveys show that most people are willing to choose a proenvironmental travel mode when they are traveling a short distance and that they are more concerned about the efficiency and travel cost over a longer distance. Consequently, if the quality of the public transportation service (speed, punctuality, comfort, and accessibility) is satisfactory even at peak hours, it has the potential to enhance the proportion of proenvironmental travel. Therefore, various strategies under the guidance of the public transit priority strategy, BRT, subsidies for public transportation, and bicycle sharing systems all stimulate proenvironmental travel. Although the promoting effects may be different for different individuals, they help to create a premise for proenvironmental travel.

The biggest challenge in promoting proenvironmental travel is how to make people who own a private car reduce their car use as much as possible. At present in China, both the family income and the private car ownership rate are undergoing a period of rapid growth. People have strong material consumption values at this stage. Additionally, there is a dual difficulty for the whole society in promoting proenvironmental travel. Many people without cars tend to choose a proenvironmental mode, but their travel mode choice may change once they have a car as the car ownership will change the situation of the travel decision. With an increase in the percentage of private car travel, the roads will become more crowded and the public transport service quality will decrease rapidly. Consequently, some people will gradually abandon public transport again. This will form a vicious circle, which can only be broken when people choose a proenvironmental travel mode based on their attitudes.

However, according to the surveys, men with a high income who travel for business have a closer correlation with carbon-intensive travel, while women with a medium income accept proenvironmental travel modes relatively easily. Changing the travel mode of the men with a high income needs a more powerful influence of social norms and the elimination of material consumption values. As a matter of fact, more and more researchers are focusing on how to educate and intervene in people's decisions to reduce car use and choose a proenvironmental travel mode.

## Figures and Tables

**Table 1:** The variables and variable value assignment.

Characteristics	Variables/code	Variable assignment
Personal	Gender/G	G = 1, male; G = 2, female
	Age/Ag	Ag = 1, when ≤20; Ag = 2, when (20, 50]; Ag = 3, when >50
	Occupation/O	O = 1~7, civil servant, manager, technician, student, staff, freelancer, others
	Monthly income/M	M = 1, when <2000; M = 2, when ≥2000
	Driver's license/J	J = 1, no; J = 2, yes
	Bus ID card/P	P = 1, no; P = 2, yes

Family-owned private travel tool	Private car/S	S = 1, no; S = 2, yes
	Bicycle/Z	Z = 1, no; Z = 2, yes
	Electric bicycle/D	D = 1, no; D = 2 when = 1; D = 3 when ≥2

Travel	Purpose/MD	MD = 1~4, commuting, business, shopping and recreation, visiting friends and relatives
	Cost/FY	FY = 1~4, ≤2 Yuan, (2, 5] Yuan, (5, 10] Yuan, >10 Yuan
	Time/SJ	SJ = 1~4, <10 minutes, 10~30 minutes, 30~60 minutes, >1 h
	Distance/JL	JL = 1~4, ≤1 km, (1, 5] km, (5, 10] km, >10 km

**Table 2:** The calculated parameters of the MNL model.

Variable value	Walking	Bicycle	Electric bicycle	Bus	Taxi	Car
Intercept	0	−3.819	.306	3.878	4.341	6.677
[G = 1] male	0	−1.826	−1.158	−1.377	−.563	−.603
[G = 2] female	0	0^b^	0^b^	0^b^	0^b^	0^b^
[Ag = 1] (0, 20]	0	3.316	1.190	.792	4.963	5.472
[Ag = 2] (20, 50]	0	.486	−.894	−.858	−1.467	.277
[Ag = 3] (50, 100)	0	0^b^	0^b^	0^b^	0^b^	0^b^
[O = 1] civil servant	0	−.232	−19.249	−.094	−17.610	−.113
[O = 2] manager	0	2.680	2.118	3.282	3.513	2.900
[O = 3] technician	0	1.970	−.031	−.739	.191	−.953
[O = 4] student	0	−.819	−1.927	−.486	2.329	.503
[O = 5] staff	0	2.438	1.662	1.469	3.882	1.273
[O = 6] freelancer	0	2.034	−.729	.211	4.093	2.956
[O = 7] others	0	0^b^	0^b^	0^b^	0^b^	0^b^
[M = 1] <2000	0	1.479	.938	.648	−.783	−.991
[M = 2] ≥2000	0	0^b^	0^b^	0^b^	0^b^	0^b^
[J = 1] no	0	.940	.337	.885	.203	−.969
[J = 2] yes	0	0^b^	0^b^	0^b^	0^b^	0^b^
[P = 1] no	0	.499	.805	−.995	−.247	.145
[P = 2] yes	0	0^b^	0^b^	0^b^	0^b^	0^b^
[S = 1] no	0	1.367	.591	.183	.143	−3.343
[S = 2] yes	0	0^b^	0^b^	0^b^	0^b^	0^b^
[Z = 1] no	0	−3.151	.135	−.700	.115	−.273
[Z = 2] yes	0	0^b^	0^b^	0^b^	0^b^	0^b^
[D = 1] 0	0	2.142	−5.036	.591	.307	−.915
[D = 2] 1	0	2.352	.474	1.804	1.503	.812
[D = 3] 2	0	0^b^	0^b^	0^b^	0^b^	0^b^
[MD = 1] commuter	0	.311	.615	−1.765	−2.738	−3.957
[MD = 2] business	0	2.193	4.095	.392	−3.909	−.101
[MD = 3] shopping and recreation	0	−.256	.650	−1.521	−.798	−1.149
[MD = 4] visiting friends and relatives	0	0^b^	0^b^	0^b^	0^b^	0^b^
[FY = 1] ≤2 Yuan	0	−.910	−1.863	−3.866	−10.625	−7.750
[FY = 2] (2, 5] Yuan	0	1.141	2.818	1.065	−4.943	−5.508
[FY = 3] (5, 10] Yuan	0	16.380	18.516	15.654	12.968	14.204
[FY = 4] >10 Yuan	0	0^b^	0^b^	0^b^	0^b^	0^b^
[SJ = 1] ≤10 mins	0	−1.728	−2.042	−20.295	2.134	−16.459
[SJ = 2] (10, 30] mins	0	.325	1.056	1.380	3.301	4.070
[SJ = 3] (30, 60] mins	0	−1.106	−.873	.032	.183	.897
[SJ = 4] >60 mins	0	0^b^	0^b^	0^b^	0^b^	0^b^
[JL = 1] ≤1 km	0	−1.601	−1.078	−21.012	−20.712	−4.768
[JL = 2] (1, 5] km	0	−1.362	−.811	−.648	−1.557	−2.105
[JL = 3] (5, 10] km	0	1.460	1.571	.660	.896	1.188
[JL = 4] >10 km	0	0^b^	0^b^	0^b^	0^b^	0^b^

^b^Because this parameter is redundant, it is set to be zero.

**Table 3:** Comparison of predicted and observed selection.

Observed values	Predicted values						
	Walking	Bicycle	Electric bicycle	Bus	Taxi	Car	Accuracy
Walking	45	8	3	4	0	1	73.8%
Bicycle	12	26	3	6	1	1	53.1%
Electric bicycle	2	4	31	9	0	2	64.6%
Bus	5	6	5	68	4	7	71.6%
Taxi	0	2	0	3	27	4	75%
Private car	2	0	3	2	5	118	90.8%

**Table 4:** Pseudo *R*-square.

Cox & Snell	.851
Nagelkerke	.881
McFadden	.564
